# Tissue factor/FVIIa activates Bcl-2 and prevents doxorubicin-induced apoptosis in neuroblastoma cells

**DOI:** 10.1186/1471-2407-8-69

**Published:** 2008-03-06

**Authors:** Jun Fang, Lubing Gu, Ningxi Zhu, Hao Tang, Carlos S Alvarado, Muxiang Zhou

**Affiliations:** 1 Division of Hematology/Oncology, Department of Pediatrics, Emory University School of Medicine, Atlanta, USA; 2 Department of Hematology, Union Hospital, Tongji Medical College, Huazhong University of Science and Technology, Wuhan, China; 3 Department of Neurosurgery, Wuhan No.1 Hospital, Wuhan, China

## Abstract

**Background:**

Tissue factor (TF) is a transmembrane protein that acts as a receptor for activated coagulation factor VII (FVIIa), initiating the coagulation cascade. Recent studies demonstrate that expression of tumor-derived TF also mediates intracellular signaling relevant to tumor growth and apoptosis. Our present study investigates the possible mechanism by which the interaction between TF and FVIIa regulates chemotherapy resistance in neuroblastoma cell lines.

**Methods:**

Gene and siRNA transfection was used to enforce TF expression in a TF-negative neuroblastoma cell line and to silence endogenous TF expression in a TF-overexpressing neuroblastoma line, respectively. The expression of TF, Bcl-2, STAT5, and Akt as well as the phosphorylation of STAT5 and Akt in gene transfected cells or cells treated with JAK inhibitor and LY294002 were determined by Western blot assay. Tumor cell growth was determined by a clonogenic assay. Cytotoxic and apoptotic effect of doxorubicin on neuroblastoma cell lines was analyzed by WST assay and annexin-V staining (by flow cytometry) respectively.

**Results:**

Enforced expression of TF in a TF-negative neuroblastoma cell line in the presence of FVIIa induced upregulation of Bcl-2, leading to resistance to doxorubicin. Conversely, inhibition of endogenous TF expression in a TF-overexpressing neuroblastoma cell line using siRNA resulted in down-regulation of Bcl-2 and sensitization to doxorubicin-induced apoptosis. Additionally, neuroblastoma cells expressing high levels of either endogenous or transfected TF treated with FVIIa readily phosphorylated STAT5 and Akt. Using selective pharmacologic inhibitors, we demonstrated that JAK inhibitor I, but not the PI3K inhibitor LY294002, blocked the TF/FVIIa-induced upregulation of Bcl-2.

**Conclusion:**

This study shows that in neuroblastoma cell lines overexpressed TF ligated with FVIIa produced upregulation of Bcl-2 expression through the JAK/STAT5 signaling pathway, resulting in resistance to apoptosis. We surmise that this TF-FVIIa pathway may contribute, at least in part, to chemotherapy resistance in neuroblastoma.

## Background

Tissue factor (TF) is a transmembrane protein that belongs to the class II cytokine receptor superfamily that shares a significant degree of homology with the interferon gamma receptor [1]. It binds to coagulation factor VII (FVII) and its active form (FVIIa), thus initiating the coagulation cascade via the extrinsic pathway. In addition to its role in coagulation, accumulating evidence suggests that TF regulates intracellular signaling pathways [2], that play a crucial role in embryonic development [3], inflammation [4], angiogenesis [5,6], and tumor development and metastasis as well [6,7]. These latter two processes are mediated through activation of the Src family – which subsequently activates PI3K/Akt and p38 MAPK pathways to positively regulate tumor cell growth [8,9] and PAR-2 activation as well – which results in increased cell migration [10,11]. Also, phosphorylation of the TF cytoplasmic domain by palmitoylation has been found to be relevant for tumor metastasis [12]. Moreover, specific interaction of the cytoplasmic domain of TF with actin-binding protein-280 (ABP-280) has been shown to mediate tumor cell metastasis and vascular remodeling in human bladder carcinoma cells [13].

There is an increasing body of evidence demonstrating that overexpression of TF is a characteristic marker for certain neoplasms. High levels of TF expression have been observed in a variety of human cancers, namely glioma [14], breast [15], lung [16], colon [17], prostate [18], pancreas [19] and ovarian cancer [20]. In these malignancies, TF is expressed either by the tumor cells themselves or the adjacent stromal cells, and expression of TF has been shown to correlate with malignant grade, metastasis, and poor prognosis. Also, studies in mice demonstrate that TF-induced cellular signaling is involved in tumor growth and metastasis [21-25].

Despite a significant body of research on the role of TF on tumor growth and metastases in some solid tumors, the mechanisms involved in both TF-mediated signaling control of apoptosis and the cellular response to anticancer treatments has not been studied in any detail thus far. A link between TF signaling and apoptosis was first suggested by the studies of Sorensen and Versteeg et al [26,27], who demonstrated that binding of FVIIa to TF transfected into baby hamster kidney (BHK) cells protected them against serum deprivation and loss-of-adhesion induced apoptosis, primarily through induction of the PI3K/Akt and p42/p44 MAPK pathways. In addition, the FVIIa/TF complex has been shown to induce BHK cell survival by both activation of STAT5 transcription factor and upregulation of the anti-apoptotic factor Bcl-XL [28]. A recent study showed that the FVIIa/TF complex prevents apoptosis in human breast cancer [29]. Although these observations suggest that TF may play a role in regulation of cell survival and prevention of apoptosis, the mechanisms by which TF signaling exerts an anti-apoptotic function in human cancer are not well understood.

In the present study, we examined TF expression in neuroblastoma cell lines, and evaluated the role of TF/FVIIa-mediated signaling in regulating survival and apoptosis of tumor cells treated with the chemotherapeutic drug doxorubicin. We also examined the possible mechanisms by which TF/FVIIa interaction regulates anti-apoptosis in neuroblastoma cells.

## Methods

### Cell lines

Two neuroblastoma cell lines SH-EP1 and SK-N-SH were used. The SH-EP1 is a substrate-adherent (S) type clone of SK-N-SH [30]. The cell lines were cultured in RPMI 1640 supplemented with 10% fetal bovine serum (FBS), 2 mM L-glutamine, 50 units/ml penicillin, and 50 μg/ml streptomycin, at 37°C and in a humidified atmosphere containing 5% carbon dioxide.

### Plasmids and gene transfection

The full-length TF ORF containing a hemagglutinin (HA) tag at the 5' end was inserted into the pcDNA3.1 vector (Invitrogen, Carlsbad, CA) between the BamH1 and Xba1 sites to create pcDNA3.1/TF. Other constructs that express truncated TF tagged at the N-terminal end with HA, i.e. pcDNA3.1/TF (extro) containing extracellular domain and transmembrane domain and pcDNA3.1/TF (intro) containing cytoplasmic domain and transmembrane domain were derived from pcDNA3.1/TF by replacing the full-length TF ORF with corresponding PCR products that lack the nucleotides for either the last 40 or the first 256 amino acids of TF, respectively.

For the transient gene transfection assay, SK-N-SH cells were plated at a density of 1 × 10^6^/well in a 6-well plate one day before transfection. Transfection was performed with Lipofectamine 2000™ (Invitrogen, CA), according to instructions by the manufacturer. A total of 4 μg of plasmid DNA was used for transfection in each well, and the total DNA supplied was kept constant in all cases by adjusting with empty vector. The nearly confluent cells were harvested 48 h after transfection, and used in experiments analyzing gene expression and sensitivity to treatment with doxorubicin.

### SiRNA transfection

Both TF siRNA and STAT5 siRNA were used to inhibit endogenous TF and STAT5 expression, respectively. As previously reported, the sequence of TF siRNA was 5'-GCGCTTCAGGCACTACAAA-3' [31]; this was synthesized for us by Dharmacon (Chicago, IL). The STAT5 siRNA was purchased from Invitrogen. A mismatched siRNA was used as a control. Transfection of siRNA into cells was performed using siPORT™NeoFX™ Reagent (Ambion, Austin TX). Briefly, cells were trypsinized and diluted in RPMI 1640 medium supplemented with 10% FBS to a density of 1 × 10^5 ^per ml. The siRNA solution and siPORT™NeoFX™ Reagent were diluted in OPTI-MEMI media and mixed following the manufacturer's protocols, then the prepared cells were added to the plates containing these siRNA/siPORT™NeoFX™ complexes. Cells were incubated at 37°C until ready to assay. All treatments were performed in triplicate.

### Western blotting

First, whole cell protein samples were prepared by lysing cells in a buffer composed of 150 mM NaCl, 50 mMTris (pH 8.0), 5 mM EDTA, 1% (vol/vol) Nonidet P40, 1 mM phenylmethylsulfonyl fluoride (PMSF), 20 ug/mL aprotinin, and 25 ug/mL leupeptin for 30 min at 4°C. After this denaturation, equal amounts of the protein extracts were resolved by sodium dodecyl sulfate-polyacrylamide gel electrophoresis (SDS-PAGE) and transferred to a nitrocellulose membrane. After blocking the membrane with buffer containing 20 mM Tris-HCl (pH 7.5) and 500 mM NaCl-5% nonfat milk for 1 hour at room temperature, it was subsequently incubated with primary antibodies for 3 hours at room temperature, followed by washes and treatment with horseradish peroxidase-labeled secondary antibody. These blots were developed using a chemiluminescent detection system (ECL; Amersham Life Science, Buckinghamshire, England). After stripping, the membrane was re-probed with an anti-β-actin antibody to control for equal protein loading and protein integrity.

### Clonogenic assay

The soft agarose method was used to measure colony formation. Briefly, a bottom layer of low-melting point agarose solution containing 0.5% agarose in a final concentration of 1 × RPMI 1640 medium supplemented with 10% FBS and the various concentrations of test reagents such as FVIIa (American Diagnostica Inc. Stamford, CT) and siRNA, was poured into gridded 35 mm dishes and allowed to gel. The top layer contained prepared (trypsinized, siRNA transfected, counted) cells, 0.35% agarose, and the 1 × medium as diluent. The cells in soft agarose were then cultured at 37°C in a humidified atmosphere containing 5% carbon dioxide. After 1 to 2 weeks, the cultures were fixed with formalin and the colonies were scored under phase microscopy (using a cutoff value of 50 viable cells).

### Cytotoxicity and apoptosis assays

The effects of TF transfection and TF inhibition by siRNA on cell growth and apoptosis of tumor cells after treatment with doxorubicin in the presence or absence of activated FVIIa were determined using a water-soluble tetrazolium salt (WST-1) assay and the annexin-V assay, respectively. Doxorubicin concentrations used in the experiments were those achieved with therapeutic doses, and the concentration of FVIIa used (10 uM) corresponds to the normal plasma concentration of zymogen FVII. For the WST-1 assay, cells were cultured in 96-well microtiter plates along with different concentrations of doxorubicin, for 44 hr. Then, WST (25 μg/well) was added and the cells were incubated for an additional 4 hr. The final optical density (OD) in the wells was read with a microplate reader at a test wavelength of 450 nm and a reference wavelength of 620 nm.

For the annexin-V assay, tumor cells incubated with or without doxorubicin were harvested by trypsinization, washed with PBS, and then stained with FITC-annexin-V (Oncogene, San Diego, CA) and PI for 30 min according to the manufacturer's instructions. Stained cells were detected using the FACScan (Becton Dickinson) and analyzed using WinList software (Verity Software House, Inc).

## Results

### TF expression and sensitivity to doxorubicin in neuroblastoma cell lines

**Figure 1:**
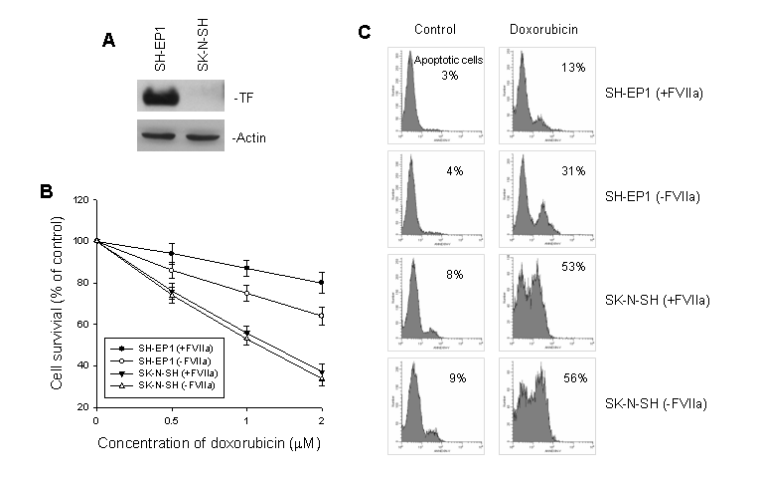
TF expression and sensitivity to doxorubicin-induced apoptosis in neuroblastoma cell lines. **A**. western blot assay for expression of TF in two neuroblastoma cell lines SH-EP1 and SK-N-SH. Actin served as a control for equal protein loading in western blotting. **B**, response to doxorubicin-induced cell death of SH-EP1 and SK-N-SH. Cells were cultured with or without 10 nM FVIIa and different concentrations of doxorubicin for 48 hrs, and cell survival was determined by WST assay. Data represent mean ± SD detected in triplicate experiments. **C**, doxorubicin-induced apoptosis in SH-EP1 and SK-N-SH. Cells were cultured with or without 10 nM FVIIa in the absence (control) or presence of 2 μM doxorubicin for 48 hrs, and apoptotic cells were detected by annexin-V staining analyzed using flow cytometry.

We have examined TF protein in two neuroblastoma cell lines and found that the level of TF expression was remarkably variable among the different cell lines. As shown in Fig. 1A, SH-EP1 cells expressed very high levels of TF, whereas SK-N-SH cells lacked TF expression as detected by western blot assay. SH-EP1 cells were much more resistant to doxorubicin than SK-N-SH cells. The difference in mean cell survival between the two cell lines after 48-hr of drug exposure was significantly greater for the SH-EP1 line in either presence or absence of FVIIa (Fig 1B). Consistent with these observations, the apoptosis assay showed a lower percentage of annexin-V positive SH-EP1 cells after 48 hr of drug treatment compared to SK-N-SH cells (13 vs. 53 %, p < 0.01) (Fig. 1C). Interestingly, for SH-EP1 cells but not for SK-N-SH cells, cell survival and apoptosis observed after doxorubicin were significantly different depending upon the presence or absence of FVIIa in the assays (80 vs. 64% for cell survival and 13 vs. 31% for apoptosis in Fig. 1B and 1C, respectively, p < 0.05).

### Effect of inhibiting TF by siRNA on doxorubicin-induced apoptosis in SH-EP1 cells

**Figure 2:**
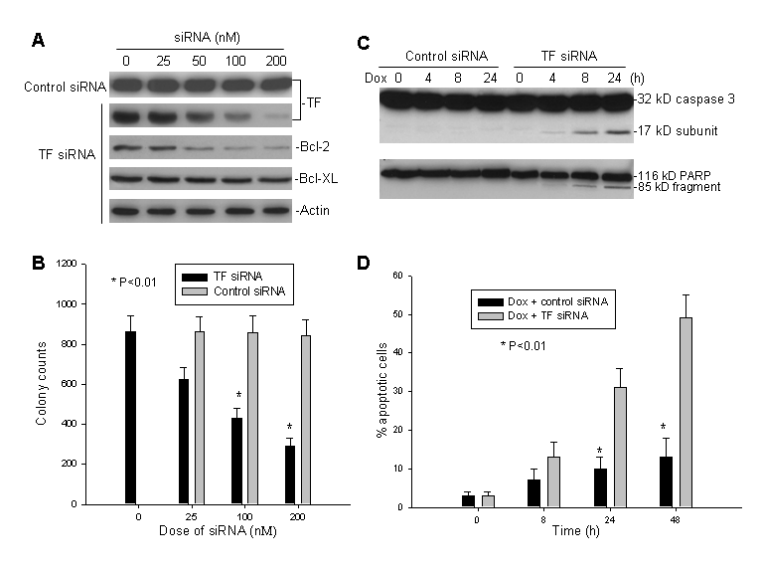
The effects of TF silencing by siRNA on the expression of endogenous TF, Bcl-2 and Bcl-XL as well as cell growth and apoptosis induced by doxorubicin in SH-EP1. **A**, cells cultured with 10 nM FVIIa were treated with different concentrations of either TF siRNA or control siRNA for 24 h. Expression of endogenous TF, Bcl-2 and Bcl-XL was detected by western blot assay. **B**, clonogenic assay of SH-EP1 in culture with TF siRNA. Cells (1 × 10^4^) were cultured in soft agarose in the presence of FVIIa for 2 weeks with different doses of TF siRNA and control siRNA, respectively, and resulting colonies counted. Data for the colony counts is shown (mean ± S.D. for triplicate cultures). **C, **western blot assay for activation of caspase-3 and cleavage of the death substrate PARP in SH-EP1 cells that were treated with 2 μM doxorubicin (Dox) and 200 nM of either TF siRNA or control siRNA for the indicated time points. **D. **quantitative detection of apoptotic cells induced by Dox when given in combination with either TF siRNA or control siRNA. SH-EP1 cells were treated with Dox (2 μM) plus siRNA (200 nM) for the indicated times, and apoptotic cells were detected by flow cytometry. Data represents the percentage of annexin-V positive cells.

To examine whether silencing of endogenous TF by TF siRNA had any effect on Bcl-2 and Bcl-XL expression and resistance of SH-EP1 cells to doxorubicin, we used a specific TF siRNA shown to effectively inhibit TF expression in a previous report [31]. SH-EP-1 cells were cultured in medium with 10 nM FVIIa for all experiments. As seen in Fig. 2A, transfection of TF siRNA into high-expressor SH-EP1 cells effectively downregulated expression of TF as well as Bcl-2 in a dose-dependent manner. Furthermore, inhibition of the endogenous TF by TF siRNA suppressed SH-EP1 cell growth, as compared with control siRNA (Fig. 2B). Also, transfection of TF siRNA sensitized SH-EP1 cells to doxorubicin-induced apoptosis, as measured both activation of caspase-3 and cleavage of the death substrate PARP. As demonstrated in Fig. 2C, significant cleavage of caspase-3 and PARP was detected in TF siRNA-transfected cells 8 hours after doxorubicin treatment, whereas cleavage of these two proteins was not observed in the control siRNA-treated cells even after 24 hours of doxorubicin treatment. Likewise, in the quantitative apoptosis assay, 24 hours post-treatment with doxorubicin and TF siRNA, there was an increased percentage of annexin-V positive SH-EP1 cells compared to that observed after doxorubicin plus control siRNA treatment (Fig. 2D).

### Effect of TF/FVIIa on Bcl-2 expression and sensitivity to doxorubicin in TF-transfected cells

**Figure 3:**
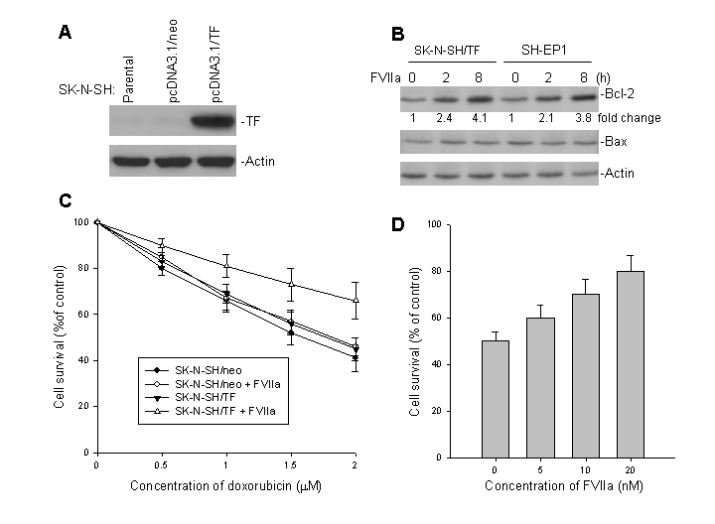
Transfection of the TF gene and its effect on Bcl-2 expression and sensitivity to doxorubicin. **A**, SK-N-SH cells lacking TF expression were transfected with the expression plasmid pcDNA3.1/TF and control plasmid (an empty neo pcDNA3.1 plasmid). Expression of transfected TF was detected by western blot assay. **B**, the effect of interaction between TF and FVIIa on Bcl-2 and Bax expression. SK-N-SH transfected with TF and SH-EP1 expressing endogenous TF were both incubated with 10 nM FVIIa for the indicated time points. Cell extracts were then analyzed for Bcl-2 and Bax expression by western blot. Labels under Bcl-2 bands in the blot represent expression levels after normalization for actin, compared with untreated (0) samples (defined as 1 unit) **C**, effect of enforced TF expression on cell survival of SK-N-SH cells in the presence or absence of FVIIa in response to doxorubicin. Cells transfected with TF and control cells (1 × 10^4^) were treated with or without 10 nM FVIIa and different doses of doxorubicin. Cells were incubated for 48 h, then cell viability was determined by WST assay. **D**, a similar WST assay of dose-dependent inhibition of FVIIa on doxorubicin-induced cell death. SK-N-SH cells transfected with TF were treated with 2 μM doxorubicin in the presence or absence of different concentrations of FVIIa as indicated.

To further explore the role of TF expression in apoptosis, expression of Bcl-2 and sensitivity to doxorubicin was evaluated in TF-transfected SK-N-SH cells in the presence of FVIIa. TF was efficiently transfected and expressed in SK-N-SH cells (Fig. 3A). FVIIa treatment of either TF-transfected SK-N-SH cells or endogenously- TF expressing SH-EP1 cells significantly induced Bcl-2 expression, whereas expression of Bax (a member of the Bcl-2 family) remained unchanged (Fig. 3B). To investigate whether induction of Bcl-2 by TF/FVIIa can affect sensitivity to doxorubicin-induced cell death, we performed a WST cytotoxicity assay in TF-transfected-SK-N-SH cells treated with FVIIa. As shown in Fig. 3C, transfection of TF alone into SK-N-SH cells did not increase resistance to doxorubicin. However, in presence of FVIIa resistance of these transfected cells to doxorubicin was significantly enhanced in an FVIIa dose-dependent manner (Fig. 3D).

### Induction of STAT5 and Akt by TF/FVIIa interaction

**Figure 4:**
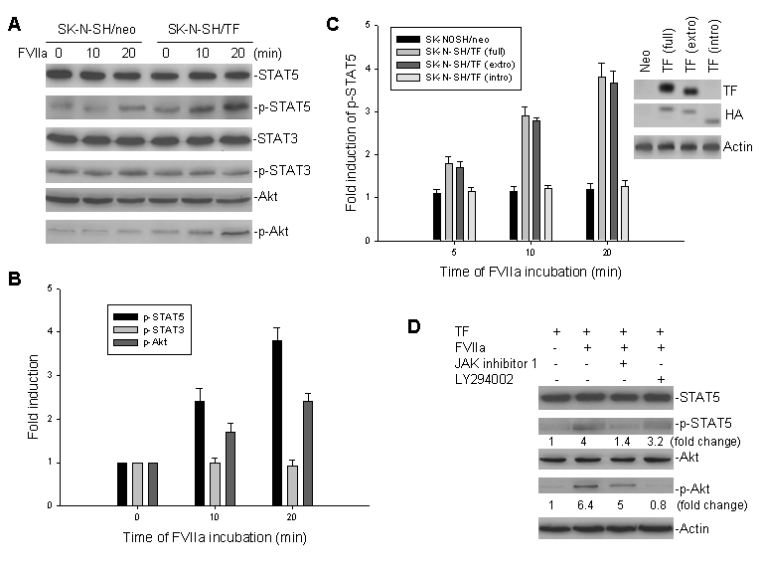
Activation of STAT5 and Akt by TF/FVIIa interaction. **A**, western blot assays for expression of STAT5, STAT3 and Akt, as well as their phosphorylated products (p-STAT5, pSTAT3 and p-Akt) in TF-transfected SK-N-SH cells treated with FVIIa. Cells were incubated with or without 10 nM FVIIa for 10 min and 20 min respectively, and then cell extracts were analyzed for protein expression as indicated. **B**, graphical representation of the relative protein levels of p-STAT5, p-STAT3, and p-Akt compared to non-phosphorylated proteins in SK-N-SH/TF cells treated with FVIIa. Data are representatives of at least three independent experiments. **C**, phosphorylation of STAT5 by the interaction between FVIIa and either full-length TF or different truncated TF proteins. SK-N-SH cells transfected with different plasmids as indicated were incubated with 10 nM FVIIa for different time. Phosphorylation of STAT5 was detected by western blot assay. Data shown as the fold induction of mean (± SD) p-STAT5 levels from at least three independent experiments, compared to controls. The expression of either TF or HA (tag) in SK-N-SH cells transfected with different TF plasmids was detected by western blot (*insert*). The TF mAb (TF9-10H10, Calbiochem) only recognizes the extracellular domain of TF. **D**, Blockage of TF/FVIIa-induced phosphorylation of STAT5 and Akt by specific JAK and PI3K inhibitors, respectively. TF-transfected SK-N-SH cells were incubated with or without 10 nM FVIIa, along with either 600 nM JAK inhibitor 1 or 20 μM of the PI3K inhibitor LY294002 as indicated for 20 min. Induction of p-STAT5 and p-Akt as well as expression of STAT5 and Akt were detected by western blot assay.

Since the JAK/STAT5 and PI3k/Akt signaling pathways have been reported to be involved in regulation of cell survival mediated by the TF/FVIIa interaction, we tested the phosphorylation of STAT5 (p-STAT5) and Akt (p-Akt) in TF-transfected SK-N-SH cells treated with FVIIa. FVIIa treatment induced phosphorylation of STAT5, which was detectable 5 min post-addition of FVIIa and reached a maximum effect 20 min thereafter (Fig. 4A and 4B). Similarly, induction of Akt phosphorylation was detected only in TF-transfected but not control-transfected SK-N-SH cells. Expression of pan-STAT5 and pan-Akt was not affected by FVIIa, suggesting that the observed increases in phosphorylation of STAT5 and Akt were most likely due to active phosphorylation of existent molecules, but not a product of upregulation.

In order to investigate whether the cytoplasmic domain of TF is involved in activation of STAT5, two expression plasmids containing a cytoplasmic domain deletion [pcDNA3.1/TF (extro)] and only the cytoplasmic domain of TF [pcDNA3.1/TF (intro)] were tested. In presence of FVIIa, transfection of the pcDNA3.1/TF (extro) lacking the cytoplasmic domain into SK-N-SH cells was able to induce phosphorylation of STAT5 comparable to that in cells transfected with the plasmid containing the full-length TF. In contrast, transfection of pcDNA3.1/TF (intro) under similar stimulation of FVIIa did not induce STAT5 phosphorylation (Fig. 4C). These results indicate that the cytoplasmic tail of TF is not the part of the molecule involved in phosphorylation of STAT5.

We also performed similar experiments of TF-transfected cells to study the effect of stimulation with FVIIa in the presence of the specific JAK inhibitor 1 and PI3k inhibitor LY294002, in order to examine whether activation of STAT5 and Akt were altered, respectively. As shown in Fig. 4D, JAK inhibitor 1 did block phosphorylation of STAT5, whereas LY294002 inhibited Akt phosphorylation in TF-transfected SK-N-SH cells treated with FVIIa. These results suggest that both pathways are also involved downstream of the TF/FVIIa interaction in human cells and can be blocked by specific pathway inhibitors.

### TF/FVIIa interaction-induced Bcl-2 expression via the JAK/STAT5 signaling pathway

**Figure 5:**
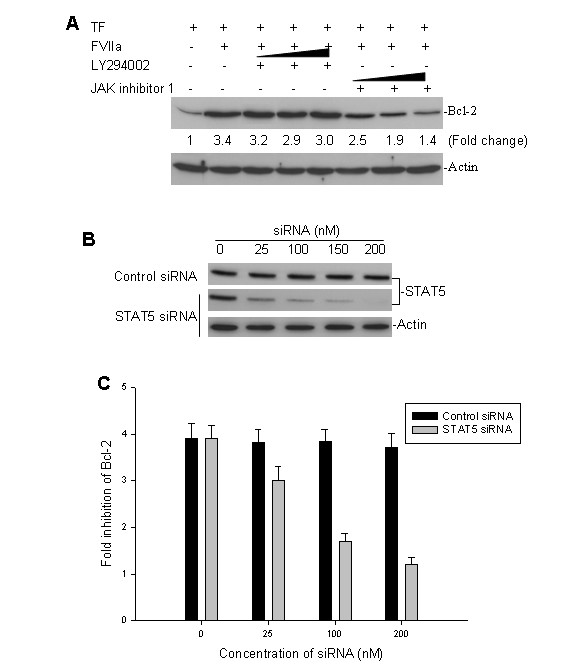
Effects of inhibiting STAT5 on TF/FVIIa-induced Bcl-2 expression. **A**, TF-transfected SK-N-SH cells were incubated with or without 10 nM FVIIa, and elevating doses of JAK inhibitor 1 (100, 300, 600 nM) and LY294002 (5, 10, 20 μM) for 2 h. Bcl-2 expression was detected by western blot assay. **B**, TF-transfected SK-N-SH cells were treated with different concentrations of either STAT5 siRNA or control siRNA for 24 h. The expression of endogenous STAT5 was detected by western blot analysis. **C**, TF-transfected SK-N-SH cells were incubated with 10 nM FVIIa in the absence or presence of varying doses of either STAT5 siRNA or control siRNA. Bcl-2 expression was detected by western blot, and densitometric data for the Bcl-2 bands provides the degree of inhibition of TF/FVII-induced Bcl-2 expression achieved by STAT5 siRNA treatment. The western blots shown in Figs A and B are representative of at least three independent experiments, and data shown in Fig C are mean fold inhibition (± SD) of Bcl-2 of three independent assays.

Since we found that TF/FVIIa interaction induces the JAK/STAT5 and PI3K/Akt survival signaling pathways as well as the anti-apoptotic factor Bcl-2, we evaluated whether there are associations between Bcl-2 induction and either induction of STAT5 or Akt activation. We tested for the expression of Bcl-2 in FVIIa-stimulated, TF-transfected SK-N-SH cells in the presence of either JAK inhibitor 1 or LY294002. As shown in Fig. 5A, we found that JAK inhibitor 1, but not LY294002, blocked the TF/FVIIa-induced upregulation of Bcl-2. This JAK inhibitor 1-mediated suppression of Bcl-2 was dose-dependent. Our results imply that TF/FVIIa-upregulation of Bcl-2 is mediated by the JAK/STAT5 pathway.

To further prove that the JAK/STAT5 pathway contributes to TF/FVIIa-induced upregulation of Bcl-2, we also tested for Bcl-2 expression in FVIIa-stimulated, TF-transfected SK-N-SH cells, in which the STAT5 was silenced by a prior transfection of STAT5 siRNA. As can be seen in Fig 5B, transfection of the STAT5 siRNA inhibited the expression of endogenous STAT5 expression, and consequently blocked TF/FVIIa-mediated upregulation of Bcl-2 (Fig. 5C). This appears to suggest that not only JAK, but also STAT5 is involved in the upregulation of Bcl-2 following the interaction of FVIIa with TF.

## Discussion

In this study, we demonstrate that the coagulation factor TF is distinctly expressed in varying degrees in human neuroblastoma cells. Thus, one of the cell lines, SH-EP1, expressed extremely high levels of TF, whereas the SK-N-SH cell line completely lacked expression of this protein. This study also demonstrates that downregulation of TF in SH-EP1 cells by siRNA sensitized these cells to doxorubicin-induced apoptosis. Conversely, transfection of TF into the TF-negative tumor cell line SK-N-SH caused resistance to doxorubicin and increased cell survival.

Enforced expression of TF alone was not sufficient to enhance resistance to doxorubicin. However, after stimulation with FVIIa, the TF-transfected cells became significanlty resistant to doxorubicin. These results suggest that the observed TF-mediated response to chemotherapeutic drugs is dependent upon its ligation by FVIIa. Another significant finding in this study was that FVIIa and tumor-expressed TF interaction induced Bcl-2 expression, an anti-apoptotic protein regulated by the JAK/STAT5 signaling pathway.

It has been previously demonstrated that the interaction between TF and FVIIa induces many intracellular signaling events involved in apoptosis, such as activation of the PI3K/Akt and JAK/STAT5 pathways [8,28]. Consistent with these observations, both Akt and STAT5 were activated (i.e. phosphorylated) in our TF-transfected cells in the presence of FVIIa. The use of pharmacological inhibitors revealed that only inhibition of the JAK/STAT5 signaling pathway blocked TF/FVIIa-induced Bcl-2 expression. Inhibition of PI3K antagonized activation of Akt, but failed to repress TF/FVIIa-induced Bcl-2 expression. These results clearly suggest that the TF/FVIIa interaction induces Bcl-2 expression only through JAK/STAT5, but not the PI3K/Akt pathway.

STAT5, a member of the signal transducers and activators of transcription (STAT) family, mediates a pro-survival signal for cytokine- and growth factor-induced cell survival, and thus it has an anti-apoptotic effect [32]. Cytokine receptors such as the interferon-gamma receptor recruit the members of the cytoplasmic Janus kinase (JAK) family, which subsequently mediates recruitment and activation of STAT5 [33]. The TF protein shares considerable homology with the cytokine receptor class II family, including the interferon gamma receptor. Thus, TF has the potential to activate STAT5 through JAK when regulating the gene expression of its targets. Normal transcriptional targets of STAT5 are known to include Bcl-2. Also, previous studies showed that the Bcl-2 gene promoter contains a STAT5 consensus element, and that STAT5 is able to induce Bcl-2 expression [34,35]. In contrast, downregulation of Bcl-2 was detected in STAT5-deficient mice and bone marrow cells, even when stimulated with SCF and IL-3 [36,37]. Our results using siRNA to knock down endogenous STAT5, did indeed inhibit TF/VFIIa-induced upregulation of Bcl-2, further confirming STAT5 regulatory role of Bcl-2 expression.

This study, also examined the role played by the cytoplasmic domain of TF in the observed upregulation of Bcl-2 by TF/FVIIa. Previous studies on the role of the cytoplasmic domain of TF in regulating cellular signaling have reported conflicting results. While some studies suggest that the intracellular domain of TF is required to confer full pro-metastatic potential to this protein [12,23,38]; other studies have concluded that the TF cytoplasmic tail is not involved in TF/FVIIa-mediated intracellular signaling [28,39]. Our results support the notion that the cytoplasmic domain of TF is not necessary for activation of STAT5/Bcl-2 pathway.

## Conclusion

Neuroblastoma, the most common extracranial solid tumor in children, is a biological heterogeneous tumor that exhibits a wide range of clinical behavior, with some tumors undergoing spontaneous regression or maturation, while others exhibit an aggressive growth and have a high metastatic potential. Most of the latter tumors are intrinsically resistant or become resistant to chemotherapy and patients have a poor prognosis. The results of this in vitro study in neuroblastoma cell lines suggest that TF overexpression may be an additional biological factor that determines an aggressive tumor phenotype and poor treatment outcome for the patients. The study also provides some insight into how to block TF activation of Bcl-2, a finding which may have therapeutic implications.

## Competing interests

The author(s) declare that they have no competing interests.

## Authors' contributions

JF and MZ designed the research and wrote the paper. MZ also provided financial support for the study. LG, NZ and HT participated and assisted with the experiments. CSA assisted with writing of the manuscript. All authors read and approved the final manuscript.

## Pre-publication history

The pre-publication history for this paper can be accessed here:



## References

[B1] Bazan JF (1990). Structural design and molecular evolution of a cytokine receptor superfamily. Proc Natl Acad Sci USA.

[B2] Spek CA (2004). Tissue factor: from 'just one of the coagulation factors' to a major player in physiology. Blood Coagul Fibrinolysis.

[B3] Pedersen B, Holscher T, Sato Y, Pawlinski R, Mackman N (2005). A balance between tissue factor and tissue factor pathway inhibitor is required for embryonic development and hemostasis in adult mice. Blood.

[B4] Chu AJ (2005). Tissue factor mediates inflammation. Arch Biochem Biophys.

[B5] Chen J, Bierhaus A, Schiekofer S, Andrassy M, Chen B, Stern DM, Nawroth PP (2001). Tissue factor – a receptor involved in the control of cellular properties, including angiogenesis. Thromb Haemost.

[B6] Belting M, Ahamed J, Ruf W (2005). Signaling of the tissue factor coagulation pathway in angiogenesis and cancer. Arterioscler Thromb Vasc Biol.

[B7] Versteeg HH, Spek CA, Peppelenbosch MP, Richel DJ (2004). Tissue factor and cancer metastasis: the role of intracellular and extracellular signaling pathways. Mol Med.

[B8] Versteeg HH, Hoedemaeker I, Diks SH, Stam JC, Spaargaren M, van Bergen En Henegouwen PM, van Deventer SJ, Peppelenbosch MP (2000). Factor VIIa/tissue factor-induced signaling via activation of Src-like kinases, phosphatidylinositol 3-kinase, and Rac. J Biol Chem.

[B9] Peppelenbosch MP, Versteeg HH (2001). Cell biology of tissue factor, an unusual member of the cytokine receptor family. Trends Cardiovasc Med.

[B10] Camerer E, Huang W, Coughlin SR (2000). Tissue factor- and factor X-dependent activation of protease-activated receptor 2 by factor VIIa. Proc Natl Acad Sci USA.

[B11] Hjortoe GM, Petersen LC, Albrektsen T, Sorensen BB, Norby PL, Mandal SK, Pendurthi UR, Rao LV (2004). Tissue factor-factor VIIa-specific up-regulation of IL-8 expression in MDA-MB-231 cells is mediated by PAR-2 and results in increased cell migration. Blood.

[B12] Dorfleutner A, Ruf W (2003). Regulation of tissue factor cytoplasmic domain phosphorylation by palmitoylation. Blood.

[B13] Ott I, Fischer EG, Miyagi Y, Mueller BM, Ruf W (1998). A role for tissue factor in cell adhesion and migration mediated by interaction with actin-binding protein 280. J Cell Biol.

[B14] Hamada K, Kuratsu J, Saitoh Y, Takeshima H, Nishi T, Ushio Y (1996). Expression of tissue factor correlates with grade of malignancy in human glioma. Cancer.

[B15] Ueno T, Toi M, Koike M, Nakamura S, Tominaga T (2000). Tissue factor expression in breast cancer tissues: its correlation with prognosis and plasma concentration. Br J Cancer.

[B16] Sawada M, Miyake S, Ohdama S, Matsubara O, Masuda S, Yakumaru K, Yoshizawa Y (1999). Expression of tissue factor in non-small-cell lung cancers and its relationship to metastasis. Br J Cancer.

[B17] Seto S, Onodera H, Kaido T, Yoshikawa A, Ishigami S, Arii S, Imamura M (2000). Tissue factor expression in human colorectal carcinoma: correlation with hepatic metastasis and impact on prognosis. Cancer.

[B18] Akashi T, Furuya Y, Ohta S, Fuse H (2003). Tissue factor expression and prognosis in patients with metastatic prostate cancer. Urology.

[B19] Kakkar AK, Lemoine NR, Scully MF, Tebbutt S, Williamson RC (1995). Tissue factor expression correlates with histological grade in human pancreatic cancer. Br J Surg.

[B20] Han LY, Landen CN, Kamat AA, Lopez A, Bender DP, Mueller P, Schmandt R, Gershenson DM, Sood AK (2006). Preoperative serum tissue factor levels are an independent prognostic factor in patients with ovarian carcinoma. Clin Oncol.

[B21] Zhang Y, Deng Y, Luther T, Muller M, Ziegler R, Waldherr R, Stern DM, Nawroth PP (1994). Tissue factor controls the balance of angiogenic and antiangiogenic properties of tumor cells in mice. J Clin Invest.

[B22] Abe K, Shoji M, Chen J, Bierhaus A, Danave I, Micko C, Casper K, Dillehay DL, Nawroth PP, Rickles FR (1999). Regulation of vascular endothelial growth factor production and angiogenesis by the cytoplasmic tail of tissue factor. Proc Natl Acad Sci USA.

[B23] Bromberg ME, Sundaram R, Homer RJ, Garen A, Konigsberg WH (1999). Role of tissue factor in metastasis: functions of the cytoplasmic and extracellular domains of the molecule. Thromb Haemost.

[B24] Mueller BM, Ruf W (1998). Requirement for binding of catalytically active factor VIIa in tissue factor dependent experimental metastasis. J Clin Invest.

[B25] Kakkar AK, Chinswangwatanakul V, Lemoine NR, Tebbutt S, Williamson RC (1999). Role of tissue factor expression on tumour cell invasion and growth of experimental pancreatic adenocarcinoma. Br J Surg.

[B26] Sorensen BB, Rao LV, Tornehave D, Gammeltoft S, Petersen LC (2003). Antiapoptotic effect of coagulation factor VIIa. Blood.

[B27] Versteeg HH, Spek CA, Richel DJ, Peppelenbosch MP (2004). Coagulation factors VIIa and Xa inhibit apoptosis and anoikis. Oncogene.

[B28] Versteeg HH, Spek CA, Slofstra SH, Diks SH, Richel DJ, Peppelenbosch MP (2004). FVIIa:TF induces cell survival via G12/G13-dependent Jak/STAT activation and BclXL production. Circ Res.

[B29] Jiang X, Guo YL, Bromberg ME (2006). Formation of tissue factor-factor VIIa-factor Xa complex prevents apoptosis in human breast cancer cells. Thromb Haemost.

[B30] Ciccarone V, Spengler BA, Meyers MB, Biedler JL, Ross RA (1989). Phenotypic diversification in human neuroblastoma cells: expression of distinct neural crest lineages. Cancer Res.

[B31] Holen T, Amarzguioui M, Wiiger MT, Babaie E, Prydz H (2002). Positional effects of short interfering RNAs targeting the human coagulation trigger tissue factor. Nucleic Acids Res.

[B32] Debierre-Grockiego F (2004). Anti-apoptotic role of STAT5 in haematopoietic cells and in the pathogenesis of malignancies. Apoptosis.

[B33] Ihle JN (1995). The Janus protein tyrosine kinase family and its role in cytokine signaling. Adv Immunol.

[B34] Weber-Nordt RM, Egen C, Wehinger J, Ludwig W, Gouilleux-Gruart V, Mertelsmann R, Finke J (1996). Constitutive activation of STAT proteins in primary lymphoid and myeloid leukemia cells and in Epstein-Barr virus (EBV)-related lymphoma cell lines. Blood.

[B35] Buitenhuis M, Baltus B, Lammers JW, Coffer PJ, Koenderman L (2003). Signal transducer and activator of transcription 5a (STAT5a) is required for eosinophil differentiation of human cord blood-derived CD34 + cells. Blood.

[B36] Snow JW, Abraham N, Ma MC, Bronson SK, Goldsmith MA (2003). Transgenic bcl-2 is not sufficient to rescue all hematolymphoid defects in STAT5A/5B-deficient mice. Exp Hematol.

[B37] Shelburne CP, McCoy ME, Piekorz R, Sexl V, Roh KH, Jacobs-Helber SM, Gillespie SR, Bailey DP, Mirmonsef P, Mann MN, Kashyap M, Wright HV, Chong HJ, Bouton LA, Barnstein B, Ramirez CD, Bunting KD, Sawyer S, Lantz CS, Ryan JJ (2003). Stat5 expression is critical for mast cell development and survival. Blood.

[B38] Belting M, Dorrell MI, Sandgren S, Aguilar E, Ahamed J, Dorfleutner A, Carmeliet P, Mueller BM, Friedlander M, Ruf W (2004). Regulation of angiogenesis by tissue factor cytoplasmic domain signaling. Nat Med.

[B39] Versteeg HH, Sorensen BB, Slofstra SH, Van den Brande JH, Stam JC, van Bergen en Henegouwen PM, Richel DJ, Petersen LC, Peppelenbosch MP (2002). VIIa/tissue factor interaction results in a tissue factor cytoplasmic domain-independent activation of protein synthesis, p70, and p90 S6 kinase phosphorylation. J Biol Chem.

